# Hepatitis C antibody prevalence and behavioral correlates in people who inject drugs attending harm reduction services in Lisbon, Portugal

**DOI:** 10.3389/fpubh.2022.952909

**Published:** 2022-08-23

**Authors:** Adriana Curado, Paulo Jorge Nogueira, Ana Virgolino, João Santa Maria, Luís Mendão, Cristina Furtado, Francisco Antunes

**Affiliations:** ^1^Instituto de Saúde Ambiental, Faculdade de Medicina, Universidade de Lisboa, Lisboa, Portugal; ^2^Laboratório Associado TERRA, Faculdade de Medicina, Universidade de Lisboa, Lisboa, Portugal; ^3^Grupo de Ativistas em Tratamentos, Lisboa, Portugal; ^4^Laboratório de Biomatemática, Faculdade de Medicina, Universidade de Lisboa, Lisboa, Portugal; ^5^Instituto Nacional de Saúde Doutor Ricardo Jorge, Lisboa, Portugal

**Keywords:** hepatitis C virus, injecting drug use, prevalence, behavioral correlates of infection, Portugal

## Abstract

The hepatitis C virus (HCV) infection is an important public health problem, affecting millions of people worldwide. People who inject drugs (PWID) are at increased risk of HCV infection due to, among other factors, widespread unsafe injecting practices, such as sharing of infected equipment or unprotected sexual practices. In Portugal, there is a lack of data regarding the proportion of infected persons through injecting drug use. This study aimed to evaluate the anti-HCV prevalence and behavioral correlates of infection in PWID attending harm reduction services in the Metropolitan Area of Lisbon, Portugal. A cross-sectional study with a purposive sample of PWID was undertaken between March 2018 and March 2020. Participants were recruited through the harm-reduction services of a nongovernmental organization. A rapid diagnostic test for anti-HCV screening was performed. Data on drug consumption history and current practices, past HCV testing, care and treatment history, and knowledge regarding hepatitis C were also collected through a questionnaire applied by trained inquirers. A total of 176 PWID participated in this study. An overall prevalence of 70.5% of anti-HCV positive in this population was found. Those with an anti-HCV positive testing result tended to start consuming at a younger age and have a higher consumption of benzodiazepines in the last 30 days. Sharing needles and other injecting material is a frequent risk behavior among this group. Also, they are more likely to have attended an opioid agonist treatment and to have undertaken previous hepatitis C and HIV tests in the past. This study represents an important effort to better understand the HCV prevalence and behavioral correlates of infection among PWID in Portugal, as well as to better estimate those in need of HCV treatment.

## Introduction

Hepatitis C virus (HCV) infection is a current public health problem. Those who are chronically infected can have significant long-term health consequences, namely, liver fibrosis, cirrhosis, and hepatocellular carcinoma (HCC) (1, 2). People who inject drugs (PWID) or have performed so in the past have often high rates of HCV infection (3–7). The high prevalence of infection in Europe among PWID is mainly due to widespread unsafe injecting practices, such as sharing of infected equipment. Furthermore, a large proportion of acute infections are attributable to injecting drug use, which accounts for 60% of the cases (8). In Western Europe, the estimated HCV antibody prevalence among PWID is around 53.2% (9). In Portugal, however, the number of people living with chronic HCV infection, as well as the proportion of infected through injecting drug use, is uncertain. The only exception is for PWID enrolled in abstinence-based programs or for cases of acute HCV infections. In 2019, the estimated HCV prevalence among PWID in the country ranged from 54 to 88% (10).

Current PWID, as well as people with an injecting history, even though brief, are frequently unaware of their HCV status. This is in part because the initial phases of HCV infection are often asymptomatic (3, 4). Other reasons are the significant barriers PWID can face when accessing HCV testing and care services (11, 12). The European Monitoring Centre for Drugs and Drug Addiction (EMCDDA) recently published a checklist specifying the potential barriers to HCV testing at the system, provider, and client levels (13). Some of the most common problems identified in the European context are the lack of knowledge and awareness of HCV infection among healthcare providers and clients, social marginalization and stigmatization of PWID, and inadequate regulations of HCV testing and care in community settings (11, 12, 14, 15).

In 2016, the World Health Organization (WHO) set the goal of eliminating viral hepatitis as a major public health threat by 2030, defined as a reduction in 90% of the incidence of infections and 65% in mortality compared with the 2015 baseline (16). The treatment of HCV is improving as direct-acting antiviral (DAA) regimens have a short duration with higher rates of sustained virologic response (SVR) and fewer serious adverse events than previous treatment options. People living with HCV should be treated regardless of disease stage or ongoing substance use (17, 18). In 2015, Portugal adopted universal access to HCV treatment (19), although PWID uptake seems to continue suboptimal. HCV treatment remains mostly hospital-based, with a few community-based experiences (20).

To accelerate progress toward hepatitis C elimination, strong efforts are needed to reach those undiagnosed or at higher risk of being infected. Implementation of HCV testing and linkage to care requires large-scale coordination efforts, innovation, and resources. A comprehensive combination of harm reduction interventions, such as Needle and Syringe Programs and Opioid Agonist Treatment, has proven to decrease virus transmission and contribute to a decline in future liver disease-related morbidity (21–25). Harm reduction also plays an important role in promoting continuity of care by linking PWID to all sorts of health services, including HCV specialized care and treatment. Increasing treatment capacity means more and more providing HCV treatment in community settings, such as primary healthcare centers and harm reduction services, to improve access to vulnerable populations (16, 25–27).

In Portugal, there is limited epidemiological data available on HCV infection among PWID. For this reason, more studies are needed to characterize the current situation in settings that are key to reach vulnerable populations, as is the case of harm reduction services. Against this background, this study aimed to evaluate the HCV prevalence and behavioral correlates of infection among PWID attending harm reduction services in the Metropolitan Area of Lisbon, Portugal.

## Materials and methods

This study followed a cross-sectional design with a purposive sample of PWID. Data collection was carried out between March 2018 and March 2020.

### Study participants and sampling

Participants were recruited through harm-reduction services of a non-governmental organization (NGO) and its partners in the Metropolitan Area of Lisbon, within their regular initiatives targeting PWID. Recruiting venues included a fixed location harm reduction center, a mobile drug consumption room, and outreach testing (using a mobile unit) in the vicinity of open drug scenes in the Metropolitan Area of Lisbon.

Services had to fulfill the following eligibility criteria to participate: (i) having a needle exchange program running; (ii) having a private room/space for data collection; (iii) having at least one trained person available to be responsible for data collection (including the application of the point-of-care test to the participants). Having a previous history of anti-HCV positivity was not an excluding criterion.

Individuals attending one of the recruiting venues were systematically invited to participate in this study. Inclusion criteria for the enrollment in this study were as follows: (i) attending to a needle and syringe exchange program; (ii) being 18 years of age or more; (iii) reporting injecting drug use in the past 30 days; (iv) not being enrolled in a drug treatment program requiring abstinence; (v) speaking Portuguese; and (vi) providing oral consent to participate in this study. Individuals with psychological disorders which could hinder their ability to give informed consent to participate were excluded.

### Study procedures

Data collection was performed by trained inquirers, who were peer workers (trained current or former PWID working in the harm-reduction services), health professionals, or social workers. The training happened before the start of data collection and covered the following areas: (i) routes of HCV transmission, prevention, and treatment; (ii) general information about the study, namely, goals, methods for data collection, procedures for the collection of the informed consent before participants' involvement, and data registration; (iii) how to apply the questionnaire; (iv) how to perform the anti-HCV point-of-care tests; and (v) procedures for referring anti-HCV positive participants.

Data collection took place between March 2018 and March 2020. The initial estimated minimum sample size was between 171 and 384 individuals. These sample size values were calculated considering a worst-case scenario prevalence of 50% [compatible with some literature that points out to a 40–50% HCV prevalence (28)], considering 7.5 and 5% absolute errors for the respective estimate of 95% confidence interval.

The invitation to participate was performed in the collaborating harm-reduction services. After the invitation, to those who met the inclusion criteria and agreed to participate, an informed consent form was handed out by the inquirers. Later, a rapid HCV antibody test was performed. The refusal to be screened for HCV would invalidate participation in this study. While waiting for the test results, trained harm-reduction workers applied a face-to-face interviewer-administered questionnaire on drug consumption and history of risk behaviors to the participant. Each questionnaire was assigned to a unique alphanumeric identification code by the inquirer to assure data anonymity and confidentiality.

Whenever the screening test result was invalid, the test was performed again, after the participant's agreement. To those with an anti-HCV reactive test, linkage to a reference hospital in the Metropolitan Area of Lisbon was offered.

### Instruments and outcomes

The questionnaire covered the following six main areas: (i) sociodemographic information; (ii) drug consumption and needle/syringe sharing; (iii) risk factors for HCV and HIV; (iv) past HCV testing, care, and treatment history; (v) knowledge regarding hepatitis C (treatment and prevention); and (vi) the results of the HVC testing. The questionnaire either in its original language (Portuguese) or a free translation to English is presented in Supplementary materials 1, 2.

### Statistical analysis

Descriptive statistics were used to present individual variables, absolute and relative frequencies for categorical variables, and mean, median, and standard deviations for numerical variables. A one-sample chi-square test was used to calculate equal proportions among categories within a single variable. Additionally, a binomial confidence interval was calculated for the HCV estimated prevalence. Bivariable analyses were performed using chi-square and Fisher's exact tests for categorical variables and the *T*-test (or the respective nonparametric Mann-Whitney *U*-test) when the test variable was numeric. Prevalence ratios were also presented along with the respective 95% confidence intervals calculated using the Katz-log method. Calculations were performed using IBM SPSS Statistics for Windows, Version 26.0. Armonk, NY: IBM Corp and R (R Foundation for Statistical Computing, Vienna, Austria).

### Ethical issues

This study followed the Declaration of Helsinki principles (29) and has received approval from the Ethics Committee of the Centro Académico de Medicina de Lisboa (Ref.ª 396/17) and from the Comissão de Ética para a Saúde (Ref.ª 514/2017). This study has also received a positive appreciation from the Portuguese National Data Protection Commission (Ref.ª 12516/2017). Only those PWID who agreed to participate and gave oral informed consent took part in this study. To address the difficulties arising from the process of obtaining informed consent, especially from traditionally high-risk groups such as PWID (i.e., assure an informed and voluntary decision to participate) and protect their interests (30, 31), a written informed consent document explaining the study objectives, data collection, analysis and storage procedures, and data confidentiality and anonymity was read out loud by the inquirer to the participant. The inquirer marked the participant's agreement in a form collected by the research team. The chosen procedure was approved by both Ethics Committees that assessed the project.

## Results

A total of 176 PWID participated in this study, being observed with a prevalence of anti-HCV positive of 70.5% (Table 1). Of the participating PWIDs (both with anti-HCV positive and negative testing), 158 (89.8%) were male. Participants had a mean age of 42.08 ± 0.63 years, with 56.8% being over 40 years of age. Anti-HCV positivity was 68.4% among younger PWID and 72.0% among older PWID. Regarding the country of origin, 149 (84.7%) participants were from Portugal, while a minority was from African Portuguese-Speaking Countries or from Northern and Eastern Europe countries, being observed a difference between both groups (the percentage of participants who tested positive for the anti-HCV is higher among those born in Portugal). In terms of the level of education, more than half of the participants had 9 years of education or less. In the 12 months before the inquiry, more than half (57.1%) of the respondents were homeless.

**Table 1:** Sociodemographic characteristics of the participants.

	**HCV+**	***p-value***	**PR**
	**% (95% CI)**		**Ratio (95% CI)**
All subjects (*n =* 176)	70.5 (63.1–77.1)	–	–
Gender		0.107^†^	
Male (*n =* 158; 89.8%)	68.4 (60.5–75.5)		1.00
Female (*n =* 15; 8.5%)	93.3 (68.1–99.8)		1.37 (1.15–1.62)
Transgender (*n =* 3; 1.7%)	66.7 (9.4–99.2)		0.98 (0.44–2.19)
Age		0.621^††^	
≤40y (*n =* 76; 43.2%)	68.4 (56.7–78.6)		1.00
>40y (*n =* 100; 56.8%)	72.0 (62.1–80.5)		1.05 (0.87–1.28)
Mean (SD) (42.08; 0.63)	42.66 (8.41)	0.276^†††^	—-
Median (IQR) (41.00; 10.00)	42.00 (10.00)		
Minimum–maximum (23–81)	28–81		
Country of birth		<0.001^††††^	
Portugal (*n =* 149; 84.7%)	72.5 (64.6–79.5)		1.00
Other (*n =* 27; 15.3%)	59.3 (38.8–77.6)		0.82 (0.59–1.13)
Level of education		1.000^†^	
≤9 years of school (*n =* 126; 72.0%)	70.6 (61.9–78.4)		1.00
>9 years of school (*n =* 49; 28.0%)	71.4 (56.7–83.4)		1.01 (0.82–1.25)
Homelessness ^a^ (last 12 months)		0.615^†^	
Yes (*n =* 100; 57.1%)	69.0 (59.0–77.9)		1.00
No (*n =* 75; 42.9%)	73.3 (61.9–82.9)		1.06 (0.88–1.28)

CI, confidence interval; HCV+, anti-hepatitis C virus-positive; IQR, interquartile range; PR, prevalence ratio; SD, standard deviation.

The sum of some variable responses may not be equal to No. due to missing values.

a Homelessness, person who lived in a rented room, pension, shelter, squat, streets, or car.

† Fisher's exact test;

†† chi-square test;

††† Student's t-test;

†††† one-sample chi-square test.

The mean age of the first injection differed between groups, with those with an anti-HCV positive testing result starting to consume at a younger age. Likewise, the percentage of PWID with anti-HCV positive consuming benzodiazepines in the last 30 days is higher than among those with an anti-HCV negative. The prevalence of anti-HCV positivity in users of benzodiazepines was 37% higher than in heroin users. Considering the frequency of drug injection daily, no significant differences were observed between the groups. Regarding needle sharing, this risk behavior was more frequent among those PWID who tested positive for the anti-HCV, happening more frequently in this group either ever or in the last 30 days. Consumers who reported never having shared needles and syringes have a 27% lower prevalence of testing anti-HCV positivity than those who reported having shared needles and syringes. The same happens for the anti-HCV positive group regarding the exchange of other injecting equipment. Almost half of the participants have already been in prison, but among those who have been in prison, only 30 injected drugs and only 17 exchanged injecting material, with no significant differences being observed between the two groups. Of the total of acquired syringes in the last 30 days, in a day, on average, more than seven were acquired. More than 80% of the participating PWID already attended an opioid agonist treatment and from these, 66% did it in the last 30 days. Considering the two groups analyzed, the percentage of people who tested anti-HCV positive who attended such programs is higher than those who tested anti-HCV negative (Table 2).

**Table 2:** Drug consumption, and needle and syringe sharing.

	**No. (%) or mean (SD)**	**HCV+**	***p-value***	**PR**
		**No. (%) or mean (SD)**		**Ratio** **(95% CI)**
Age of first injection				
Mean (SD)	23.19 (8.54)	21.58 (7.57)	0.001^††^	—-
Drugs injected in the last 30 days ^a^^,^^b^				
Heroin	146 (40.7)	101 (69.2)	0.513^†^	1.00
Powder cocaine	112 (31.2)	81 (72.3)	0.496^†^	1.05 (0.89–1.22)
Crack cocaine	77 (21.4)	59 (76.6)	0.135^†^	1.11 (0.94–1.31)
Benzodiazepines	18 (5.0)	17 (94.4)	0.026^†^	1.37 (1.17–1.60)
Methadone	4 (1.1)	3 (75.0)	1.000^†^	1.08 (0.61–1.93)
Other	2 (0.6)	2 (100.0)	1.000^†^	1.45 (1.30–1.61)
Frequency of drug injection: daily injection			0.320^†^	
Yes	96 (54.5)	71 (74.0)		1.00
No	80 (45.5)	53 (66.3)		0.9 (0.74–1.09)
Frequency of drug injection: >5 injections per day			0.124^†^	
Yes	63 (35.8)	49 (77.8)		1.00
No	113 (64.2)	75 (66.4)		0.85 (0.71–1.03)
Sharing of needles and syringes (ever)			0.001^†^	
Yes	58 (33.3)	50 (86.2)		1.00
No	116 (66.7)	73 (62.9)		0.73 (0.61–0.87)
Sharing of needles and syringes (last 30 days)			0.006^†^	
Yes	10 (17.2)	10 (100.0)		1.00
No	46 (79.3)	40 (87.0)		0.87 (0.78–0.97)
Does not know	2 (3.4)	–		0.00 (0.00–2.76)
Sharing of other injecting equipment (e.g., cup, filter, water, pad) (ever)				
Yes	88 (51.5)	69 (78.4)	0.062^†^	1.00
No	83 (48.5)	54 (65.1)		0.83 (0.68–1.01)
Sharing of other injecting material (e.g., cup, filter, water, pad) (last 30 days)			0.124^†^	
Yes	113 (64.2)	75 (66.4)		1.00
No	63 (35.8)	49 (77.8)		1.17 (0.97–1.41)
Ever been in prison			1.000^†^	
Yes	84 (48.6)	59 (70.2)		1.00
No	89 (51.4)	62 (69.7)		0.99 (0.82–1.21)
Injecting drug use in prison (ever)				
Yes	30 (37.5)	21 (70.0)	1.000^†^	1.00
No	50 (62.5)	35 (70.0)		1.00 (0.74–1.34)
Sharing of injecting equipment in prison			0.403^†^	
Yes	17 (58.6)	14 (82.4)		1.00
No	12 (41.4)	8 (66.7)		0.81 (0.51–1.28)
New syringes acquired by day (average) – last 30 days			0.216^†^	
Mean (SD)	7.64 (9.79)	8.25 (10.27)		—-
Opioid agonist treatment (ever)			<0.001^†^	
Yes	145 (82.9)	111 (76.6)		1.00
No	30 (17.1)	12 (40.0)		0.52 (0.33–0.82)
Opioid agonist treatment (last 30 days)			0.597^†^	
Yes	116 (82.9)	90 (77.6)		1.00
No	24 (17.1)	17 (70.8)		0.91 (0.69–1.20)

HCV+, anti-hepatitis C virus-positive; PR, prevalence ratio; SD, standard deviation.

The sum of some variable responses may not be equal to No. due to missing values.

a Methamphetamines, amphetamines, and buprenorphine with zero cases not represented in the table.

b Multiple choice questions.

† Fisher's exact test;

†† , Student's t-test.

Overall, respondents had more than two sexual partners in the last 12 months (Table 3). Moreover, also considering the last year, only 18 of the respondents had sexual relations in exchange for money or drugs. In terms of having sexual intercourse with a condom, although more than half of the respondents used a condom in the last sexual intercourse, 71 did not. In contrast, almost one-third had a piercing or tattoo performed without disposable material. No differences were observed between those who tested positive and those who tested negative for the anti-HCV test for the variables presented in Table 3.

**Table 3:** Other risk factors for anti-HCV/anti-HIV.

	**No. (%) or mean (SD)**	**HCV+**	***p-value***	**PR**
		**No. (%) or mean (SD)**		**Ratio (95% CI)**
Sexual partners in the last 12 months				
Mean (SD)	2.19 (3.87)	2.07 (4.06)	0.527^††^	—-
Exchange of sex for money or drugs in the last 12 months			0.593^†^	
Yes	18 (10.5)	14 (77.8)		1.00
No	154 (89.5)	108 (70.1)		0.90 (0.69–1.18)
Use of condom at last sexual intercourse			0.374^†^	
Yes	93 (56.0)	68 (73.1)		1.00
No	71 (42.8)	47 (66.2)		0.91 (0.74–1.11)
No answer	2 (1.2)	2 (100.0)		1.37 (1.21–1.55)
Piercing or tattooing done without disposable material			0.208^†^	
Yes	53 (30.8)	41 (77.4)		1.00
No	119 (69.2)	80 (67.2)		0.87 (0.72–1.05)

HCV+, anti-hepatitis C virus-positive; PR, prevalence ratio; SD, standard deviation.

The sum of some variable responses may not be equal to No. due to missing values.

† Fisher's exact test;

†† Student's t-test.

Table 4 shows the past HCV testing, care, and treatment history of the interviewed participants. Regarding hepatitis C, the majority (87.4%) had already previously been tested. There are, however, differences between the two groups considered. In fact, 76.5% of those who were previously tested for HCV had an anti-HCV positive test. The prevalence of anti-HCV positivity in users who did not test for anti-HCV in the last 12 months is nine times higher than among those who did. Considering the last testing for HCV, only four PWID who tested positive for HCV were not aware of their previous serological status, reporting to have had a negative result in the last HCV test. Of the participants who were already previously infected with HCV, only 42 (25.5%) had received treatment for it. Of this, only 31 concluded the treatment with success, and among those who did not conclude the treatment with success, only seven were currently being followed in a center of hepatitis reference. Of the inquired participants, the majority (152) had already taken the HIV test in the past. Of these, 75.7% had an anti-HCV positive test. Among the 107 respondents who reported to have had a negative result in the last HIV test, 75 (70.1) had an anti-HCV positive test. To be highlighted the fact that among those PWID who self-reported positive status for HIV, 36 (92.3%) had also a positive anti-HCV positive test. Considering those who were referred to have had a positive result in the HIV test in the past, 34 (87.2%) are currently in HIV treatment.

**Table 4:** Past anti-HCV testing, care, and treatment history.

	**No. (%)**	**HCV+**	***p-value***	**PR**
		**No. (%) or mean (SD)**		**Ratio (95% CI)**
Ever tested for anti-HCV			<0.001^†^	
Yes	153 (87.4)	117 (76.5)		1.00
No	19 (10.9)	5 (26.3)		0.34 (0.16–0.73)
Does not know	3 (1.7)	1 (33.3)		0.44 (0.09–2.16)
Tested for anti–HCV in the last 12 months			0.140^†^	
Yes	40 (88.9)	23 (57.5)		1.00
No	5 (11.1)	5 (100.0)		1.74 (1.33–2.27)
Last HCV test result (self-reported)			<0.001^†^	
Negative	37 (25.5)	4 (10.8)		1.00
Positive	108 (74.5)	108 (100.0)		9.25 (3.67–23.34)
Ever been in treatment for HCV			—-	
Yes	42 (25.5)	42 (100.0)		1.00
No	65 (74.5)	65 (100.0)		1.00 (0.97–1.04)
Treatment concluded with success			—-	
Yes	31 (73.8)	31 (100.0)		1.00
No	10 (23.8)	10 (100.0)		1.00 (0.83–1.12)
Does not know	1 (2.4)	1 (100.0)		1.00 (0.07–3.61)
Currently followed in hospital – treatment not successful			—-	
Yes	7 (70.0)	7 (100.0)		1.00
No	3 (30.0)	3 (100.0)		1.00 (0.38–2.60)
Ever tested for anti–HIV			<0.001^†^	
Yes	152 (86.9)	115 (75.7)		1.00
No	19 (10.9)	6 (31.6)		0.42 (0.21–0.81)
Does not know	4 (2.3)	2 (50.0)		0.66 (0.25–1.77)
Last anti–HIV test result (self–reported)			0.024^†^	
Negative	107 (71.8)	75 (70.1)		1.00
Positive	39 (26.2)	36 (92.3)		1.32 (1.13–1.54)
Does not know	3 (2.0)	2 (66.7)		0.95 (0.42–2.14)
Currently in treatment (if anti–HIV positive)			1.000^†^	
Yes	34 (87.2)	31 (91.2)		1.00
No	5 (12.8)	5 (100.0)		1.10 (0.99–1.22)

a HCV+, anti-hepatitis C virus-positive; PR, prevalence ratio.

The sum of some variable responses may not be equal to No. due to missing values.

† Fisher's exact test.

Knowledge regarding HCV infection prevention and treatment was assessed with three questions. The answers to the knowledge questions are summarized in Table 5. For the three questions, a high percentage of correct responses was observed. Overall, 92.0% of the participants knew that HCV can be transmitted by sharing syringes or other equipment, 84.6% knew that condoms can be used to prevent HCV transmission, and 88.6% believed that an effective hepatitis C treatment is available. No differences were observed between anti-HCV positive and anti-HCV negative.

**Table 5:** Knowledge regarding hepatitis C (treatment and prevention).

	**No. (%)**	**HCV+**	***p-value***	**PR**
		**No. (%)**		**Ratio (95% CI)**
Mode of transmission of HCV (sharing of syringe or other equipment)			0.150^†^	
True ^a^	161 (92.0)	117 (72.7)		1.00
False	2 (1.1)	1 (50.0)		0.69 (0.17–2.76)
Does not know	12 (6.9)	6 (50.0)		0.69 (0.39–1.22)
Condom as way to prevent HCV transmission			0.075^†^	
True ^a^	148 (84.6)	108 (73.0)		1.00 (0.73–1.38)
False	15 (8.6)	11 (73.3)		0.57 (0.29–1.12)
Does not know	12 (6.9)	5 (41.7)		
Availability of effective treatment for HCV			0.150^†^	
True ^a^	155 (88.6)	113 (72.9)		1.00
False	4 (2.3)	3 (75.0)		1.03 (0.58–1.83)
Does not know	16 (9.1)	8 (50.0)		0.69 (0.42–1.13)

HCV+, anti-hepatitis C virus-positive; PR, prevalence ratio.

a Correct answer.

The sum of some variable responses may not be equal to No. due to missing values.

† Fisher's exact test.

**Figure 1:**
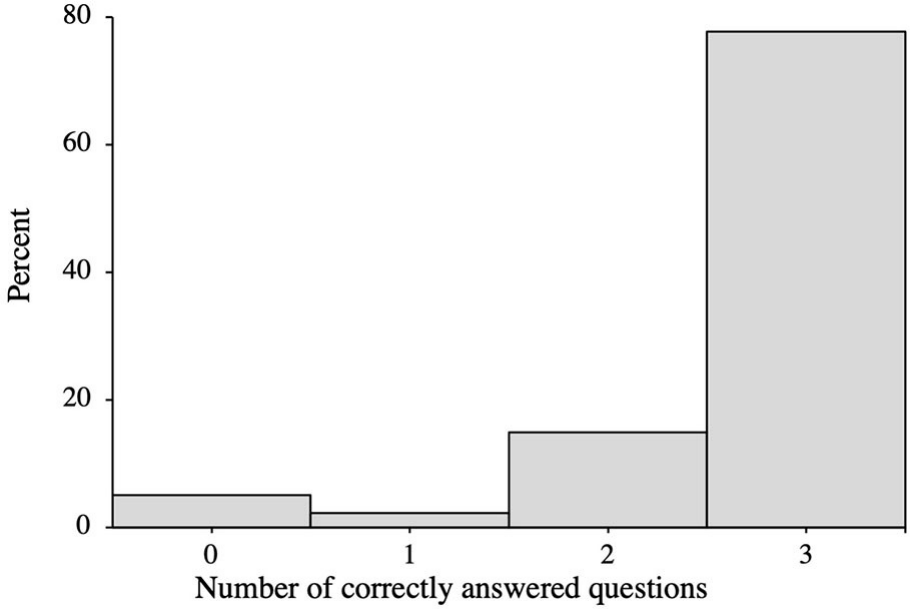
Knowledge regarding hepatitis C (treatment and prevention).

The median number of correctly answered questions was three, with a mean of 2.65 ± 0.76. The distribution of HCV knowledge scores is found in Figure 1. No statistically significant differences were found between the two groups regarding the number of correctly answered questions.

## Discussion

The main goal of this study was to evaluate the anti-HCV positive prevalence and the behavioral correlates of infection in current PWID attending harm reduction services in the Metropolitan Area of Lisbon, Portugal. This is the first study of its kind undertaken in Portugal with this at-risk population for HCV.

Data revealed a prevalence of anti-HCV positive of 70.5% among PWID. Although there is a lack of data in Portugal concerning the total number of people living with HCV among PWID [PWID account for 2.1 per 1.000 Portuguese inhabitants, according to 2015 data (32)], our results are in line with a 2018 Portuguese estimate, which indicates a high prevalence in this population [88.45% of PWID had HCV antibodies (33)]. The overall scenario in most EU/EEA countries is likewise worrisome attending to the high HCV seroprevalence among PWID (>50%) (34). Against this background, it is worth emphasizing the work performed within harm reduction programs and treatment with DAAs, which may have contributed to reduce the percentage of transmission in many EU/EEA countries (35).

In this study, the mean age of the participants who had an anti-HCV positive testing result was of 42.66 years, observing a slightly higher (non-significant) percentage of anti-HCV positivity among PWID over 40 years of age. Moreover, among the men in our sample, which represent nearly 90% of the participants in this study, more than half also tested positive for the anti-HCV. This is consistent with data from the European Centre for Disease Prevention and Control, which indicates that, across Europe, HCV is an infection predominantly affecting men aged 25–44 years (34). The higher prevalence found in PWID of older age (above 40 years of age) is supported by other recent studies, which concluded that this age group is more likely to have been exposed to HCV given their injecting practices for a longer period (36). Another significant finding of this study was the lower mean age of the first injection among those who had an anti-HCV positive test. In Western Europe, starting to inject at younger ages (under 25 years old) has been associated with an increased risk of HCV and HIV infections (37).

Heroin was the most frequently reported drug injected in the previous 30 days, along with cocaine (powder and crack). Heroin and other opioids remain, in general, in Europe, the most common primary injected drugs among treatment entrants, with a few exceptions in some European countries [for example, in the Czech Republic and Norway, methamphetamines, and amphetamines, respectively, are the most injected drugs (38)]. Apart from these substances, it is worth emphasizing the higher use of benzodiazepines among those with an anti-HCV positive test. Although the total number of people who reported to have injected benzodiazepines in the previous 30 days was low (only 18 out of 176), this reflects the tendency that has been observed in other studies of an increase in the injection of benzodiazepines among PWID (39). This new trend, among PWID, has potential critical implications, as benzodiazepines injection use has been associated (more frequently than in other injected drugs) with memory loss and disorientation, which can lead to higher-risk injecting practices, such as the use of injecting equipment of other users potentially infected with HCV.

In our sample, sharing needles and syringes was another risk behavior for HCV infection, being found a higher proportion of persons with an anti-HCV positivity among those who have previously shared that material. According to the EMCDDA (38), more than 10% of all treatment entrants who report injecting drugs have recently shared a needle or syringe. The same data indicate that the proportion of PWIDs reporting sharing used needles/syringes in the previous 4 weeks was higher in countries in Eastern Europe (47% in Bulgaria, 40% in Romania, and 39% in Hungary).

Our study found that most parts of the participants had received opioid agonist treatment in the last 30 days and that, from these, a large part had an anti-HCV positive test. Although those treatment programs are the gold standard in HCV and HIV prevention, which is in line with the projections from the EMCDDA which estimates that coverage of opioid agonist treatment in Portugal is predictably above the 2020 WHO target of 40% (38), we found in our study a higher risk for HCV infection for those in opioid agonist treatment. One possible explanation could be that our sample is small, entailing PWID with longer consumption paths and low variability among participants. Also, it is important to highlight that the study participants were enrolled in a low threshold methadone program not requiring abstinence, thus not colliding with study admission criteria that exclude those who are abstinent while in treatment. For this reason, our aim was not to evaluate the effectiveness of methadone programs in the prevention of hepatitis C.

Among the participants, a great part had already been tested before for HCV. Of these, almost a quarter underwent the last testing in the last 12 months. Furthermore, comparing the results of the anti-HCV test result (70.5) and the self-reported results of the last anti-HCV test (74.5%), it is visible very similar numbers.

In our study, among those who were already tested for anti-HIV in the past, around three-quarters were anti-HCV positive. Likewise, almost all of those who reported having had a positive result in the anti-HIV test also had an anti-HCV positive test. These results are aligned with what is already known regarding a coinfection with HIV and HCV in populations such as those of PWID. HCV and HIV share the same transmission route, and in Europe, among HCV-infected PWID, coinfection with HIV can range from 0 to 70%, with a median of 3.9%. Particularly in Portugal, coinfection prevalence (anti-HIV prevalence among anti-HCV positive) is high (>15%) (40).

Previous studies have analyzed knowledge regarding HCV among PWID. Recent findings show that, despite some knowledge gaps mainly in younger or older PWID (41, 42), these groups are now more aware of how HCV can be transmitted and about the existing treatment options, which is being linked to more HCV treatment willingness (43, 44). Despite being used only three questions, our results go in the same direction as most of the participants have shown knowing the routes of transmission of the HCV, how the infection transmission can be prevented, and that an effective treatment for HCV is already available. This higher awareness among PWID regarding HCV infection has been pointed out to be related to increasing media coverage on this matter and the rising tendencies of HCV screening and treatment in several countries (43). In addition, although not addressed in this study, socioeconomic factors have also been associated with HCV knowledge, putting into evidence the need to target PWID in disadvantaged situations (44).

Despite the contribution of this study to advance knowledge of the epidemiology of HCV infections in PWID in Portugal, there are some limitations to consider. First, a relatively small sample was selected through a non-random sampling method. Moreover, it was not possible to have a complete record of those who refused to participate in this study, after their initial invitation. For these reasons, the results cannot be generalized to all PWID. This is related to the difficulties pointed out by other authors as barriers to participation in epidemiological studies, such as fear of lack of confidentiality and anonymity, and further stigmatization or perceived need for commitment (45, 46). Even so, it must be highlighted that the identification of participants was performed by harm-reduction services of an experienced NGO with regular initiatives targeting PWID in three different venues (fixed location harm reduction center, mobile drug consumption room, and outreach testing, using a mobile unit), which allow to more promptly identify potential participants. Second, due to the abovementioned difficulties, some subgroups (e.g., women who inject drugs) were harder to be sampled. Nonetheless, the overall results of this study do not differ from other studies in the area. Third, also due to the relatively small sample, not all behavioral correlates usually associated with HCV infection were observed as significant in this study, namely, unprotected sexual intercourse or percutaneous procedures. Fourth, only self-reported HIV test results were considered for the analysis (while for HCV, we relied on testing status) as, at the time of data collection, the HIV test was optional. The major limitation of HCV is the referral to hepatitis care and treatment. Therefore, it was not possible to obtain confirmatory infection data (RNA), which would give us a better estimate of how many PWIDs need treatment.

In summary, these findings indicate a high anti-HCV positive prevalence among PWID. Age of the first injection, sharing needles, syringes, and other injecting equipment, and frequency of opioid agonist treatment seem to be potential behavioral correlates for anti-HCV positive prevalence in the analyzed population. Screening for HCV infection and subsequent linkage to care is essential to achieving the benefits of DAAs. In Portugal, DAAs are available since 2015, but PWID still present treatment uptake rates below standards.

## Conclusion

This study represents the first estimate of the anti-HCV prevalence among PWID attending harm reduction services in Portugal. Collected data contribute to increasing current knowledge regarding this population and behavioral correlates of infection, as well as to better estimate of those in need of HCV treatment. Increasing treatment capacity should be expanded to primary care physicians, and harm-reduction services on a relationship of proximity with PWID, as they are able to treat patients with HCV, as well as continued attention to prevent further transmissions through programs that offer education and harm-reduction.

## Data availability statement

Data is available upon request to the principal investigator, FA (fantunes@medicina.ulisboa.pt).

## Ethics statement

The study was reviewed and approved by Ethics Committee of the Centro Académico de Medicina de Lisboa (Ref.ª 396/17), Comissão de Ética para a Saúde (Ref.ª 514/2017) and Portuguese National Data Protection Commission (Ref.ª 12516/2017). The participants provided their written informed consent to participate in this study.

## Author contributions

Conceptualization: AC, PN, JS, LM, CF, and FA. Methodology: AC, JS, LM, CF, and FA. Validation and formal analysis: AC and PN. Investigation, supervision, and funding acquisition: AC, JS, LM, and FA. Resources and project administration: AC and FA. Writing—original draft: AC, PN, AV, and FA. Writing—review and editing: AC, PN, AV, JS, LM, CF, and FA. Visualization: PN and AV. All authors contributed to the article and approved the submitted version.

## Funding

This study was funded by Gillead Sciences Portugal (https://www.gilead.com/utility/global-operations/europe/portugal/portugal-translation) and Fundação para a Ciência e a Tecnologia (https://www.fct.pt/), grant numbers UIDB/04295/2020 and UIDP/04295/2020. The funders were not involved in the study design, data collection, analysis, decision to publish, or preparation of the manuscript.

## Conflict of interest

The authors declare that this study was conducted in the absence of any commercial or financial relationships that could be construed as a potential conflict of interest.

## Publisher's note

All claims expressed in this article are solely those of the authors and do not necessarily represent those of their affiliated organizations, or those of the publisher, the editors and the reviewers. Any product that may be evaluated in this article, or claim that may be made by its manufacturer, is not guaranteed or endorsed by the publisher.
