# Supplementary material for: Hepatitis C antibody prevalence and behavioral correlates in people who inject drugs attending harm reduction services in Lisbon, Portugal
Source: Front Public Health. 2022 Aug 23;10:952909. doi: 10.3389/fpubh.2022.952909 (PMC9445135; doi:10.3389/fpubh.2022.952909)
Supplement: Supplementary file 1 [file Table_1.DOCX]

Supplementary Material

# Supplementary material 1

**Questionário**

**1. Informação sociodemográfica**

**1.1. Data de nascimento:**

__/ __/ ____ (dia/ mês/ ano)

**1.2. País de nascimento:**

⭘ Portugal

⭘ Outro, qual?

**1.3. Género**

⭘ Masculino

⭘ Feminino

⭘ Transgénero

**1.4. Qual o grau mais elevado de escolaridade que completou?**

⭘ Sem escolaridade

⭘ 1º Ciclo

⭘ 2º Ciclo

⭘ 3º Ciclo

⭘ Secundário

⭘ Ensino Superior

⭘ Não responde

**1.5. Nos últimos 12 meses, onde é que viveu a maior parte do tempo?**

⭘ Casa própria/arrendada

⭘ Casa de familiares/amigos

⭘ Quarto arrendado

⭘ Pensão

⭘ Abrigo/albergue

⭘ Comunidade terapêutica

⭘ Prisão

⭘ Casa ocupada

⭘ Na rua

⭘ Outro, qual?

⭘ Não responde

**2. Consumo de drogas e partilha de material**

**2.1. Com que idade injetou drogas pela primeira vez?**

|__|__| anos

⭘ Não sabe

⭘ Não responde

**2.2. Que drogas injetou nos últimos 30 dias? (pode assinalar várias opções)**

☐ Heroína

☐ Cocaína em pó

☐ Cocaína-base

☐ Anfetaminas/metanfetaminas

☐ Benzodiazepinas

☐ Buprenorfina

☐ Metadona

☐ Outras, quais?

☐ Não responde

**2.3. Nos últimos 30 dias, quantos dias consumiu por via injetada?** |

|__|__| dias

⭘ Não sabe

⭘ Não responde

**2.4. Nos últimos 30 dias, em média quantas vezes injetou por dia?**

|__|__| injeções/dia

⭘ Não sabe

⭘ Não responde

**2.5. Alguma vez na sua vida usou agulhas ou seringas que sabia ou suspeitava terem sido usadas por outra pessoa, incluindo o seu companheiro(a)?**

⭘ Sim

⭘ Não

⭘ Não sabe

⭘ Não responde

**2.5.1. Se sim, quando foi a última vez que usou agulhas ou seringas que sabia terem sido usadas por outra pessoa?**

⭘ Nos últimos 30 dias

⭘ Há mais de 30 dias

⭘ Não sabe

⭘ Não responde

**2.6. Alguma vez na sua vida usou outro material de injeção (colher/carica, filtro/algodão, caldo, água, toalhete) que sabia ou suspeitava ter sido usado por outra pessoa, incluindo o seu companheiro(a)?**

⭘ Sim

⭘ Não

⭘ Não sabe

⭘ Não responde

**2.6.1. Se sim, quando foi a última vez que usou outro material de injeção que sabia ter sido usado por outra pessoa?**

⭘ Nos últimos 30 dias

⭘ Há mais de 30 dias

⭘ Não sabe

⭘ Não responde

**2.7. Alguma vez esteve preso?**

⭘ Sim

⭘ Não

⭘ Não responde

**2.7.1. Se sim, alguma vez consumiu drogas por via injetada na prisão?**

⭘ Sim

⭘ Não

⭘ Não responde

**2.7.1.1. Se sim, alguma vez na prisão usou material de injeção (agulhas, seringas, colher/carica, filtro/algodão, caldo, água, toalhete) que sabia ou suspeitava ter sido usado por outra pessoa?**

⭘ Sim

⭘ Não

⭘ Não sabe

⭘ Não responde

**2.8. Nos últimos 30 dias, onde é que obteve seringas novas? (pode assinalar várias opções)**

☐ Equipa de rua / Unidade móvel

☐ Centro fixo (ONG)

☐ Farmácia (gratuitas)

☐ Farmácia (compradas)

☐ Centro de Saúde

☐ Equipa de tratamento

☐ De outros utilizadores (compradas)

☐ De outros utilizadores (gratuitas)

☐ Outro, qual?

☐ Não responde

**2.9. Nos últimos 30 dias, em média quantas seringas novas obteve por dia?**

|__|__| número de seringas/dia

⭘ Não sabe

⭘ Não responde

**2.10. Alguma vez esteve em programa de substituição opiácea?**

⭘ Sim

⭘ Não

⭘ Não responde

**2.10.1. Se sim, indique se esteve em programa de substituição opiácea nos últimos 30 dias.**

⭘ Sim

⭘ Não

⭘ Não responde

**3. Fatores de risco**

**3.1. Com quantos parceiros(as) teve relações sexuais nos últimos 12 meses?**

|__|__| número de parceiros

⭘ Não sabe

⭘ Não responde

**3.2. Nos últimos 12 meses, teve relações sexuais em troca de dinheiro, drogas ou outros bens?**

⭘ Sim

⭘ Não

⭘ Não responde

**3.3. Utilizou preservativo na última relação sexual com penetração?**

⭘ Sim

⭘ Não

⭘ Não sabe

⭘ Não responde

**3.4. Alguma vez fez um piercing ou tatuagem em contexto informal (rua, prisão, serviço militar ou casa de amigo) sem usar material descartável?**

⭘ Sim

⭘ Não

⭘ Não sabe

⭘ Não responde

**4. História de rastreios**

**4.1. Alguma vez fez o teste da hepatite C?**

⭘ Sim

⭘ Não

⭘ Não sabe

**4.2. Se sim, quando foi a última vez que fez o teste da hepatite C?**

__/ ____ (mês/ ano)

⭘ Não sabe

**4.3. Qual foi o resultado do último teste da hepatite C que realizou?**

⭘ Negativo

⭘ Positivo

⭘ Não sabe

**4.3.1. Se positivo, alguma vez fez tratamento para a hepatite C?**

⭘ Sim

⭘ Não

**4.3.1.1. Se sim, concluiu o tratamento com sucesso?**

⭘ Sim

⭘ Não

⭘ Não sabe

*4.4.3.1.1.1. Se não concluiu com sucesso, está atualmente a ser seguido no hospital?*

⭘ Sim

⭘ Não

**4.4. Alguma vez fez o teste de VIH?**

⭘ Sim

⭘ Não

⭘ Não sabe

**4.5. Quando foi a última vez que fez o teste de VIH?**

__/ ____ (mês/ ano)

⭘ Não sabe

**4.6. Qual foi o resultado do último teste do VIH que realizou?**

⭘ Negativo

⭘ Positivo

⭘ Não sabe

**4.6.1. Se positivo, está atualmente em tratamento?**

⭘ Sim

⭘ Não

**5. Conhecimentos sobre prevenção e tratamento da hepatite C**

Para cada uma das seguintes afirmações, por favor indique se é verdadeira ou falsa:

**5.1. A hepatite C transmite-se através da partilha de seringas e de outro material de injeção.**

⭘ Verdadeira

⭘ Falsa

⭘ Não sabe

**5.2. Usar preservativo previne a transmissão da hepatite C.**

⭘ Verdadeira

⭘ Falsa

⭘ Não sabe

**5.3. Está disponível tratamento eficaz e curativo para a hepatite C.**

⭘ Verdadeira

⭘ Falsa

⭘ Não sabe
