# Supplementary material for: Hepatitis C antibody prevalence and behavioral correlates in people who inject drugs attending harm reduction services in Lisbon, Portugal
Source: Front Public Health. 2022 Aug 23;10:952909. doi: 10.3389/fpubh.2022.952909 (PMC9445135; doi:10.3389/fpubh.2022.952909)
Supplement: Supplementary file 2 [file Table_2.DOCX]

Supplementary Material

# Supplementary material 1

**Questionnaire**

**1. Sociodemographic information**

**1.1. Date of birth:**

__/ __/ ____ (day/ month/ year)

**1.2. Country of birth:**

⭘ Portugal

⭘ Other, which?

**1.3. Gender**

⭘ Male

⭘ Female

⭘ Transgender

**1.4. What is the highest level of education you have completed?**

⭘ With no formal education

⭘ 1^st^ grade

⭘ 2^nd^ grade

⭘ 3^rd^ grade

⭘ Secondary school

⭘ Higher education

⭘ No answer

**1.5. In the last 12 months, where did you live most of your time?**

⭘ Own/rented house

⭘ House of family/friends

⭘ Rented room

⭘ Pension

⭘ Shelter/inn

⭘ Therapeutic community

⭘ Prison

⭘ Squat

⭘ Street

⭘ Other, which?

⭘ No answer

**2. Drug consumption and needle/syringe sharing**

**2.1. At what age did you inject drugs for the first time?**

|__|__| years

⭘ Does not know

⭘ No answer

**2.2. Which drugs did you inject in the last 30 days? (can tick more than one option)**

☐ Heroin

☐ Powder cocaine

☐ Crack cocaine

☐ Amphetamines/ methamphetamines

☐ Benzodiazepines

☐ Buprenorphine

☐ Methadone

☐ Others, which?

☐ No answer

**2.3. In the last 30 days, for how many days did you inject?**

|__|__| days

⭘ Does not know

⭘ No answer

**2.4. In the last 30 days, on average, how many times a day did you inject?**

|__|__| injections/day

⭘ Does not know

⭘ No answer

**2.5. Have you ever used needles or syringes that you knew or suspected to have been used by someone else, including your partner?**

⭘ Yes

⭘ No

⭘ Does not know

⭘ No answer

**2.5.1. If yes, when was the last time you used needles or syringes that you knew were used by someone else?**

⭘ In the last 30 days

⭘ More than 30 days ago

⭘ Does not know

⭘ No answer

**2.6. Have you ever used any other injection material (spoon/cap, filter/cotton, broth, water, wipe) that you knew or suspected was used by someone else, including your partner?**

⭘ Yes

⭘ No

⭘ Does not know

⭘ No answer

**2.6.1. If yes, when was the last time you used another injection material that you knew was used by someone else?**

⭘ In the last 30 days

⭘ More than 30 days ago

⭘ Does not know

⭘ No answer

**2.7. Have you ever been in prison?**

⭘ Yes

⭘ No

⭘ No answer

**2.7.1. If yes, have you ever injected drugs in prison?**

⭘ Yes

⭘ No

⭘ No answer

**2.7.1.1. If yes, did you ever use injection material in prison (needles, syringes, spoon/cap, filter/cotton, broth, water, wipe) that you knew or suspected had been used by someone else?**

⭘ Yes

⭘ No

⭘ Does not know

⭘ No answer

**2.8. In the last 30 days, where did you get new syringes? (can tick more than one option)**

☐ Street team / Mobile unit

☐ Fixed center (NGO)

☐ Pharmacy (free)

☐ Pharmacy (bought)

☐ Primary Health Center

☐ Treatment team

☐ From other users (bought)

☐ From other users (free)

☐ Other, which?

☐ No answer

**2.9. In the last 30 days, on average, how many new syringes did you get per day?**

|__|__| number of syringes/day

⭘ Does not know

⭘ No answer

**2.10. Have you ever been on an opiate substitution treatment?**

⭘ Yes

⭘ No

⭘ No answer

**2.10.1. If yes, indicate whether you were in an opioid substitution treatment in the last 30 days.**

⭘ Yes

⭘ No

⭘ No answer

**3. Risk factors**

**3.1. With how many partners did you have sex in the last 12 months?**

|__|__| number of partners

⭘ Does not know

⭘ No answer

**3.2. In the past 12 months, have you had sex in exchange for money, drugs or other goods?**

⭘ Yes

⭘ No

⭘ No answer

**3.3. Did you use a condom in the last intercourse with penetration?**

⭘ Yes

⭘ No

⭘ Does not know

⭘ No answer

**3.4. Have you ever had a piercing or tattoo in an informal context (street, prison, military service or a friend's house) without using disposable material?**

⭘ Yes

⭘ No

⭘ Does not know

⭘ No answer

**4. History of testing**

**4.1. Have you ever been tested for hepatitis C?**

⭘ Yes

⭘ No

⭘ Does not know

**4.2. If yes, when was the last time you were tested for hepatitis C?**

__/ ____ (month/ year)

⭘ Does not know

**4.3. What was the result of your last hepatitis C test?**

⭘ Negative

⭘ Positive

⭘ Does not know

**4.3.1. If positive, have you ever had treatment for hepatitis C?**

⭘ Yes

⭘ No

**4.3.1.1. If yes, did you complete the treatment with success?**

⭘ Yes

⭘ No

⭘ Does not know

*4.4.3.1.1.1. If not completed with success, are you currently being followed up in an hospital?*

⭘ Yes

⭘ No

**4.4. Have you ever been tested for HIV?**

⭘ Yes

⭘ No

⭘ Does not know

**4.5. When was the last time you were tested for HIV?**

__/ ____ (month/ year)

⭘ Does not know

**4.6. What was the result of your last HIV test?**

⭘ Negative

⭘ Positive

⭘ Does not know

**4.6.1. If positive, are you currently undergoing treatment?**

⭘ Yes

⭘ No

**5. Knowledge about hepatitis C prevention and treatment**

For each of the following statements, please indicate whether it is true or false:

**5.1. Hepatitis C is spread through sharing syringes and other injection equipment.**

⭘ True

⭘ False

⭘ Does not know

**5.2. Using a condom prevents the transmission of hepatitis C.**

⭘ True

⭘ False

⭘ Does not know

**5.3. Effective and curative treatment for hepatitis C is available.**

⭘ True

⭘ False

⭘ Does not know
